# “I Know What I Have to Do, but I Don’t Do It”: The Relationship Between Knowledge and Adherence to Hand Hygiene in Healthcare Settings

**DOI:** 10.3390/healthcare13050530

**Published:** 2025-02-28

**Authors:** Joana Silva, Cristina Antunes, Samanta Batista, José Braga, Angélica Gomes, Rosa Guedes, Anabela Barbosa, Pedro Forte, Cristina Imaginário

**Affiliations:** 1 Nursing Department of Higher School of Health, University of Trás-os-Montes and Alto Douro, 5000-801 Vila Real, Portugal or joana.fernandes.silva@ulsb.min-saude.pt (J.S.); mantunes@utad.pt (C.A.); imaginario@utad.pt (C.I.); 2 RISE-Higher School of Health, University of Trás-os-Montes and Alto Douro, 5000-801 Vila Real, Portugal; 3 Unidade Local de Saúde de Braga, 4710-453 Braga, Portugal; 4 Unidade Local de Saúde de Trás-os-Montes e Alto Douro, 5000-508 Vila Real, Portugal; palmaab@chtmad.min-saude.pt (S.B.); jcbraga@chtmad.min-saude.pt (J.B.); 5 Unidade Local de Saúde do Tâmega e Sousa, 4560-136 Penafiel, Portugal; amcgomes@ulsts.min-saude.pt (A.G.); anabela343@hotmail.com (A.B.); 6 Centro Hospitalar e Universitário de Santo António, 4050-342 Porto, Portugal; rosaguedes.cardiologia@chporto.min-saude.pt; 7 Department of Sports, Higher Institute of Educational Sciences of the Douro, 4560-708 Penafiel, Portugal; 8 Research Center for Active Living and Wellbeing, Instituto Politécnico de Bragança, 5300-252 Bragança, Portugal; 9 CI-ISCE, Higher Institute of Educational Sciences of the Douro, 4560-708 Penafiel, Portugal; 10 Department of Sports Sciences, Instituto Politécnico de Bragança, 5300-252 Bragança, Portugal; 11 Centro de Investigação em Tecnologias e Serviços de Saúde (CINTESIS@RISE), Escola Superior de Enfermagem do Porto, 4200-072 Porto, Portugal

**Keywords:** hand, hygiene, knowledge, adherence, healthcare, infection, transmission

## Abstract

**Objective:** This study aims to analyze the correlation between healthcare professionals’ knowledge of hand hygiene protocols and their actual adherence to these practices. Specifically, we investigate whether higher knowledge levels correspond to better compliance, and we examine potential influencing factors such as professional role, gender, and prior training in infection control. **Methods:** A non-probabilistic, convenience sample was composed of 51 healthcare professionals working in patient care. Data collection was conducted through a questionnaire to assess knowledge and direct observation to assess adherence to hand hygiene. **Results:** The results indicate a satisfactory level of knowledge among healthcare professionals. No statistically significant differences were observed between different professional groups regarding knowledge and adherence to hand hygiene. The average adherence rate to hand hygiene was 60.13%. No statistically significant relationship was found between healthcare professionals’ knowledge and their adherence to hand hygiene. Statistically significant differences were only found between males and females regarding the adherence rate to hand hygiene, with males showing higher adherence. **Conclusions:** Although healthcare professionals demonstrate satisfactory knowledge about hand hygiene, adherence to the practice still shows variability. Therefore, implementing continuous training programs and developing institutional policies may contribute to improving hand hygiene practices, thereby reducing the incidence of healthcare-associated infections.

## 1. Introduction

Healthcare-associated infections (HAIs) represent a persistent global challenge, profoundly affecting patient safety, healthcare costs, and overall public health. These infections not only compromise the quality of care delivered but also significantly burden the healthcare systems, contributing to increased morbidity, mortality, and economic strain [1,2]. Within this context, hand hygiene (HH) is widely acknowledged as one of the most effective, low-cost interventions to prevent the transmission of pathogenic microorganisms, playing a pivotal role in reducing HAIs and improving healthcare quality metrics [3,4].

The historical significance of hand hygiene traces back to the groundbreaking work of Ignaz Semmelweis, who demonstrated its efficacy in reducing puerperal fever in the 19th century. Subsequent advancements, including Florence Nightingale’s emphasis on sanitary conditions, have established the foundation for modern infection control practices [5,6]. Despite this wealth of evidence, adherence to hand hygiene protocols among healthcare professionals remains suboptimal, with rates often falling below 50% in critical care settings [7,8].

Barriers to effective hand hygiene are multifaceted, spanning organizational, cultural, and individual domains. Factors such as time constraints, high workloads, and limited resource access exacerbate non-compliance [9,10]. Furthermore, the interplay between knowledge and behavior has been the subject of extensive research, revealing that while healthcare professionals often possess adequate theoretical understanding, this does not always translate into consistent adherence to best practices [11,12].

Hand hygiene adherence is key in preventing healthcare-associated infections (HAIs), influenced by gender, education, professional category, and infection control training. Gender differences often reflect behavioral or cultural factors, with male healthcare workers typically showing lower compliance rates, possibly due to differing risk perceptions and socialization around hygiene practices [13,14]. Education level also plays a significant role, as higher qualifications enhance theoretical understanding, though this does not always translate into consistent adherence, highlighting a persistent gap between knowledge and behavior [15,16]. Similarly, professional roles affect compliance, with nurses often demonstrating higher adherence due to frequent patient interactions, whereas operational assistants may face unique challenges like limited training [17,18]. Regular training in infection control has been shown to improve adherence, but contextual factors such as resource limitations and workload continue to hinder consistent practice [19,20]. Addressing these dynamics through tailored interventions, continuous education, and multimodal strategies may be of higher importance in fostering a culture of compliance, enhancing patient safety, and reducing HAIs [21,22]. This study aligns with global calls for improved infection prevention measures and continuous training to bridge the gap between knowledge and practice [23,24].

Considering these challenges, the present study aims to evaluate the relationship between knowledge of protocols and adherence to hand hygiene among healthcare professionals in the internal medicine department of a hospital in northern Portugal, considering factors such as education, experience, and training. By examining this dynamic, the study seeks to identify critical gaps and propose strategies to enhance compliance, ultimately reducing HAIs and promoting safer healthcare environments. It was hypothesized that knowledge, age, education level, professional category, years of service, and training may justify hand hygiene adherence.

## 2. Materials and Methods

### 2.1. Study Design and Sample

This is a quantitative, descriptive, correlational, and cross-sectional study. The study population includes all healthcare professionals working in the internal medicine department of a hospital in northern Portugal. The exclusion criteria were professionals in management roles, those absent from duty due to medical leave, and participants who did not fully complete the questionnaire. The inclusion criteria were also established to align with the research focus and objectives: participants must be nurses, physicians, or operational assistants actively providing care within the internal medicine department where the study is conducted. Thus, the study population was of convenience. The study population consisted of 59 healthcare professionals working in the internal medicine department. All the personnel meeting the inclusion criteria (nurses, physicians, and operational assistants involved in direct patient care) were invited to participate, and 51 professionals agreed to participate, resulting in a response rate of 86.4%. Given the study’s exploratory nature, the sample size was determined based on feasibility rather than a formal power calculation. The Institutional Ethical Committee approved the research project (No. 32_2023 on 8 March 2023). All the procedures were carried out by the Declaration of Helsinki, which governs research involving human subjects.

### 2.2. Data Collection Instruments

Data were collected from the study participants using a two-step approach. First, the healthcare professionals were invited to participate through internal communication channels, and informed consent was obtained before enrolment. Second, data collection involved self-administered structured questionnaires assessing hand hygiene knowledge and direct in-person observations of adherence to hand hygiene protocols. The observations were conducted discreetly during routine clinical activities using a standardized checklist to minimize potential behavioral alterations due to awareness of being monitored. Hand hygiene adherence was assessed through structured observation conducted by a trained researcher at randomly selected times across different shifts, including morning, afternoon, and night, on weekdays and weekends. Each participant was observed on three separate occasions for at least one week between sessions. No video recordings or CCTV footage were used to collect data, ensuring compliance with ethical guidelines. The observation periods were scheduled to minimize participant awareness and reduce potential behavioral changes due to the Hawthorne effect.

Given the characteristics of this research, two data collection instruments were selected: observation and a questionnaire. Considering the study’s object, structured observation was chosen using a hand hygiene observation form from the DGS [25]. The observation focuses on the key parameters to assess adherence: the opportunity for hand hygiene, observation during caregiving activities around a patient, and the actual hand hygiene action based on the observed indications. Regarding the indications for hand hygiene, there are five options related to the moments for hygiene: “before patient contact”, “before an aseptic procedure”, “after potential exposure to blood and body fluids”, “after patient contact”, and “after contact with the patient’s surrounding environment”. Regarding the action performed by the professionals, it is possible to mark “Rub”, “Wash”, or “Not performed” and note whether gloves were used. The observation was carried out at three distinct moments for each professional, with a one-week interval between sessions.

The questionnaire was applied by the principal investigator and is designed to assess healthcare professionals’ knowledge of the primary mode of the cross-transmission of microorganisms between patients, the most common source of microorganisms responsible for HAIs, the minimum time required for alcohol-based hand antiseptic solutions (ABHSs) to reduce microbial flora, the moments recommended by the WHO for hand hygiene, and the identification of true and false statements about hand hygiene with alcohol-based solutions. It also addresses situations that should be avoided in caregiving and asks participants to select the most appropriate hand hygiene method for specific situations. This instrument also facilitates the characterization of the sample in terms of gender, age, educational level, professional category, length of service, and prior training in infection control.

It was decided that direct observation should be carried out before the completion of the questionnaire by the professionals, as their behavior regarding HH could be influenced if the questionnaire were answered beforehand. Adherence to HH was calculated by dividing the number of times the technique was performed by the total number of observed opportunities following the recommendations of the DGS. A pilot test with 10 healthcare professionals confirmed face validity.

### 2.3. Data Analysis

Inferential analysis was used to analyze the relationship between knowledge and adherence to HH across professional groups, compare knowledge and adherence to HH across professional groups, and assess the relationship with sociodemographic and professional characteristics. In this regard, the sum of correct responses related to knowledge of HH practices was calculated. Reliability was assessed using Cronbach’s alpha (0.86) for internal consistency and test–retest reliability (0.81) over two weeks, ensuring the tool’s accuracy and stability.

Assumptions for parametric tests (normality of distribution and homogeneity of variances) were also evaluated using the Shapiro–Wilk and Levene tests, respectively. It was found that not all the variables followed a normal distribution; therefore, both parametric and non-parametric tests were performed. Since the results obtained were the same, the parametric tests are presented [26].

Thus, to compare the three professional groups, the parametric ANOVA test was used. To compare two independent groups (gender and professional training) regarding quantitative variables, the independent samples *t*-test was applied, as the assumptions of normality and homogeneity of variances were also met. However, Spearmen’s correlation coefficient was used to analyze the relationship between knowledge and adherence to hand hygiene (HH) and other quantitative variables (age and length of service), as well as to analyze the relationship with an ordinal variable (educational level). The significance level accepted was 5%. A mindmap was created using the Graphviz library’s Digraph class to generate a directed graph representing the study’s results for data visualization.

## 3. Results

Table 1 presents the sample (n = 51) characteristics and distribution. Percentages were presented for sex, education level, professional category, and previous training in hand hygiene.

The results indicate a high level of knowledge among healthcare professionals regarding essential hand hygiene practices (Table 2). The majority correctly identified the key aspects, such as the need for antiseptic friction (96.2%) and hand hygiene despite glove use (96.1%). However, significant gaps were observed in knowledge about the minimum time required for alcohol-based hand antiseptic (41.2% correct) and indications for hand hygiene before certain activities, such as opening a patient’s room door (11.8% correct).

Regarding adherence, the mean hand hygiene compliance rate was 60.13% (±17.14), with no significant differences across professional categories. However, male professionals exhibited higher adherence than females (t(49) = −2.14, *p* = 0.038). No statistically significant correlation was found between knowledge levels and adherence rates (r = 0.20, *p* = 0.160).

**Figure 1:**
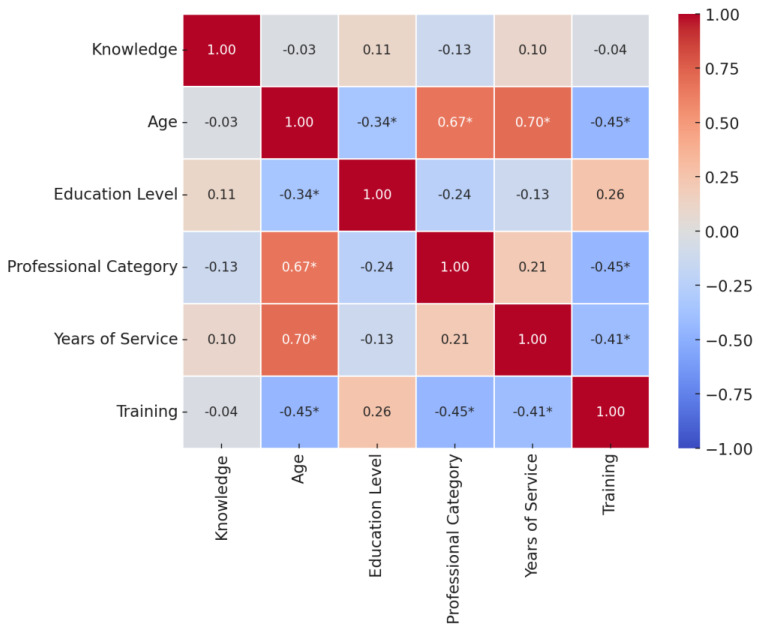
Correlations heatmap between knowledge, adherence, age, education level, professional category, years of service, and training. * *p* < 0.05.

The correlation analysis between knowledge, adherence, age, education level, professional category, years of service, and training revealed key associations. Knowledge showed a significant negative correlation with age (rs = −0.45; *p* = 0.001) and professional category (rs = −0.45; *p* = 0.001), indicating that older individuals and those in higher professional categories tend to have lower knowledge scores. Additionally, years of service were negatively associated with knowledge (rs = −0.41; *p* = 0.003), suggesting that individuals with more experience also tend to score lower in knowledge. On the other hand, education level exhibited a weak positive correlation with knowledge (rs = 0.26; *p* = 0.069), although this relationship was not statistically significant. Adherence did not show any meaningful correlation with any of the studied variables (all *p*-values > 0.05), suggesting that adherence behaviors are independent of knowledge, age, education, or experience. The correlation heatmap between knowledge, adherence, age, education level, professional category, years of service, and training is presented in Figure 1.

**Figure 2:**
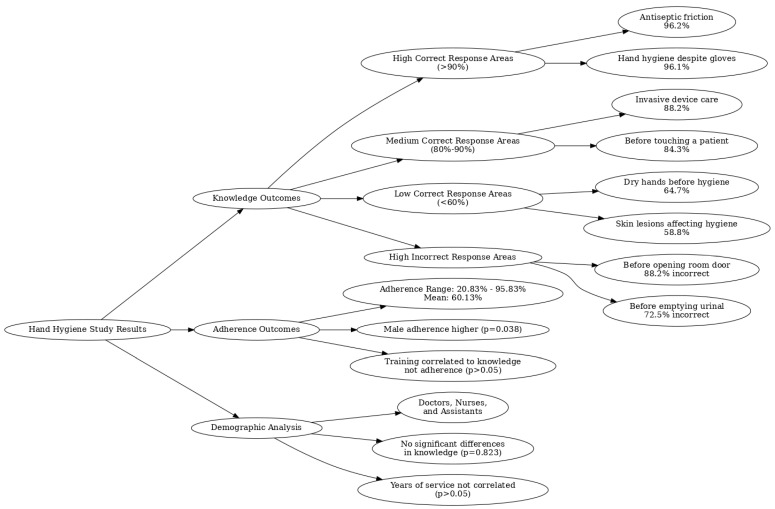
Mindmap of handwash knowledge, adherence, and demographics.

The mindmap (Figure 2) summarizes the study’s key findings, highlighting three main areas: knowledge outcomes, adherence outcomes, and demographic analysis. It illustrates the high, medium, and low correct response rates across various hand hygiene practices, with notable strengths in understanding antiseptic friction and the need for hand hygiene despite glove use, contrasted by frequent misconceptions about hand hygiene in less intuitive scenarios, such as before opening a patient’s room door. Adherence outcomes reflect variability among healthcare professionals, with an overall mean adherence rate of 60.13% and male professionals demonstrating significantly higher adherence rates. The demographic analysis reveals no significant differences in knowledge or adherence based on professional roles, years of service, or age. The visualization provides a comprehensive overview of the relationships between knowledge, adherence, and contextual factors, underscoring the areas for targeted improvement in hand hygiene practices. On average, each healthcare professional was observed during 24.0 ± 4.5 HH opportunities across the three observation sessions. The mean adherence rate was 60.13% (SD = 17.14), ranging from a minimum of 20.83% to a maximum of 95.83%. Mean adherence rates by profession are now included in the visualization for additional clarity: nurses (58.4%), physicians (61.7%), and operational assistants (59.2%).

## 4. Discussion

The study aimed to evaluate healthcare professionals’ knowledge of HH practices, analyze their adherence to these practices, and investigate the relationship between knowledge, adherence, and sociodemographic and professional variables such as age, sex, educational background, years of service, and training in infection control. The results showed that although operational assistants had a higher average of correct answers and doctors exhibited the highest adherence rate, no statistically significant differences were identified between professional groups in either knowledge or adherence.

This study reinforces previous findings indicating that high theoretical knowledge of hand hygiene does not necessarily translate into improved adherence [27,28]. Although healthcare professionals demonstrated a strong understanding of hygiene protocols, no statistically significant relationship was found between knowledge levels and compliance rates, aligning with previous studies highlighting this gap [29,30].

The observed gender differences in adherence, with male professionals demonstrating significantly higher compliance, contradict earlier findings that often report greater adherence among female healthcare workers [13,31]. This discrepancy may be influenced by workplace cultural norms or specific behavioral expectations within the study setting.

Despite ongoing efforts to improve infection control through education and training programs, our results indicate that knowledge alone is insufficient for behavioral change. Previous studies have emphasized the importance of multimodal interventions, combining education with behavior-focused strategies such as real-time feedback, environmental nudges, and institutional policies [9,23].

Additionally, while knowledge gaps were identified—particularly regarding the appropriate duration of antiseptic friction and the necessity of hand hygiene before specific tasks—these gaps do not appear to be the primary barriers to adherence. Instead, factors such as time constraints, workload, institutional enforcement, and habitual behaviors may play a more significant role in determining compliance [1,17]. Addressing these barriers requires a comprehensive approach, incorporating knowledge reinforcement, systemic improvements, workplace cultural shifts, and continuous monitoring strategies [21,32].

The relationship between sociodemographic variables, knowledge, and hand hygiene adherence was limited, with no statistically significant correlations between age, educational level, or years of service. Interestingly, professionals who participated in infection control training exhibited higher theoretical knowledge but lower adherence rates, reinforcing that knowledge alone does not guarantee adequate practices [30,33]. Additionally, it was observed that although the majority recognized the importance of HH and its relevance in the prevention of healthcare-associated infections, the average adherence rate was only 60.13%, lower than the 75.1% reported by the DGS [25]. Studies such as those by [34] and Rodrigues [32] suggest that workload, inadequate resources, and institutional barriers may compromise practical adherence, even among theoretically well-informed professionals. These findings highlight the importance of continuous educational programs, monitoring, and improvements in working conditions to promote the adoption of consistent and effective HH practices. The analysis of hand hygiene highlights the key factors influencing knowledge and adherence, including age, education level, professional category, years of service, and training. Research suggests that healthcare workers may experience a decline in knowledge over time without ongoing education or training, contributing to knowledge decay [35,36,37]. While higher education generally enhances hand hygiene knowledge, its impact varies across populations [38,39]. However, adherence appears independent of knowledge, age, and experience, as workload and environmental factors often compromise compliance [40,41,42]. These findings emphasize the need for targeted educational strategies that improve knowledge and address adherence barriers among HCWs, particularly those in senior roles. Continuous training and reinforcement of best practices remain essential to reducing healthcare-associated infections [39,43].

Despite the observed results, this study has some limitations. Although this study offers valuable insights into the relationship between knowledge and adherence to hand hygiene, its findings should be interpreted cautiously due to the relatively small sample size (n = 51). Future research should aim for larger, multicenter studies to enhance generalizability and further explore variability in adherence across different healthcare settings. The observations may cause behavior changes, inflating adherence rates. The study design does not evaluate long-term or shift-based variations. Contextual barriers like staffing or hygiene supply issues were not profoundly explored. The study uses mainly quantitative data, which is missing personal attitudes or motivations. Management roles were excluded, ignoring leadership’s influence on hygiene practices. External factors like national campaigns or policies were not considered. Future studies can improve by using large and more diverse samples across multiple hospitals and including different units such as emergency and surgical wards. Covert methods or automated tools can minimize observation bias, while longer study durations can capture trends over time and shifts. Exploring barriers like staffing, resources, and cultural norms and incorporating interviews or focus groups can provide deeper insights into personal views and motivations. Including leadership roles can highlight the impact of policies and workplace culture on hand hygiene practices. Additionally, accounting for external factors like public health campaigns and policies will offer a more comprehensive understanding of adherence and its determinants. These strategies can lead to more effective and targeted interventions.

## 5. Conclusions

This study highlights the disconnect between knowledge and practice in hand hygiene adherence among healthcare professionals. While the participants exhibited high levels of theoretical knowledge, this did not correlate with actual compliance, reinforcing that knowing what to do does not always lead to action. Addressing hand hygiene adherence requires a multifaceted approach beyond education, incorporating organizational support, behavioral interventions, and continuous monitoring to create a sustainable compliance and infection prevention culture.

## Figures and Tables

**Table 1:** Sociodemographic and professional characteristics of participants (n = 51).

Variables	n	%
Gender		
Female	42	82.4
Male	9	17.6
Educational Level		
Basic Education	2	3.9
Secondary Education (12th grade or equivalent Professional Course)	8	15.7
Bachelor’s Degree	21	41.2
Specialty	8	15.7
Master’s Degree	10	19.6
Doctorate	2	3.9
Professional Category		
Nurse	26	51
Physician	14	27.5
Operational Assistant	11	21.6
Training in Infection Control		
Yes	20	39.2
No	31	60.8

**Table 2:** Distribution of knowledge (correct or incorrect) on hand hygiene practices (n = 51).

Aspect	Correct		Incorrect	
	n	%	n	%
Antiseptic friction must cover the entire surface of the hands	49	96.2	2	3.8
Glove use exempts the need for hand hygiene	49	96.1	2	3.9
Rinse hands under running water after friction	48	94.1	3	5.9
Application of hand cream	48	94.1	3	5.9
Dry hands with a reusable towel after friction	48	94.1	3	5.9
The main mode of cross-transmission of microorganisms between patients in a care unit	47	92.2	4	7.8
Use of jewelry	47	92.2	4	7.8
Wash visibly dirty hands with water and soap	47	92.0	4	8.0
Artificial nails	46	90.2	5	9.8
Hand hygiene after maintaining invasive devices during care	45	88.2	6	11.8
Hand hygiene before touching a patient	43	84.3	8	15.7
WHO-recommended moments for hand hygiene	41	80.4	10	19.6
Hand hygiene before administering an injectable	41	80.4	10	19.6
Dry hands before performing hand hygiene	33	64.7	18	35.3
Skin lesions affecting hand hygiene	30	58.8	21	41.2
The most frequent source of microorganisms responsible for HAIs	24	47.1	27	52.9
Minimum time required for ABHS to reduce microbial flora on hands	21	41.2	30	58.8
Hand hygiene before making patient records	16	31.4	35	68.6
Friction for 5–8 min during surgical preparation	15	29.5	36	70.5
Hand hygiene before emptying a urinal	14	27.5	37	72.5
Hand hygiene before opening the patient’s room door	6	11.8	45	88.2

## Data Availability

Data will be available under the corresponding author’s contact.
